# Supplementary material for: NECAP antagonizes light-induced Rhodopsin-1 internalization to promote photoreceptor homeostasis
Source: bioRxiv. 2026 Jun 2:2026.06.01.729193. Preprint. [Version 1] doi: 10.64898/2026.06.01.729193 (PMC13252013; doi:10.64898/2026.06.01.729193)
Supplement: Supplement 1 [file NIHPP2026.06.01.729193v1-supplement-1.pdf]

bioRxiv preprint doi: https://doi.org/10.64898/2026.06.01.729193; this version posted June 2, 2026. The copyright holder for this preprint (which was not certified by peer review) is the author/funder, who has granted bioRxiv a license to display the preprint in perpetuity. It is made available under a CC-BY 4.0 International license.

ommatidia are shown in each panel. (a) *NorpA*^{EE5}*/Y* males reared under light for the indicated days. (b) *NorpA*^{EE5}*/Y; Rh1-Gal4/UAS-NECAP* flies reared under light for the indicated days. (c) *NorpA*^{EE5}*/Y* flies reared in the dark. (d) Anti-Rh1 western blot of flies of the indicated genotypes reared under light for five days. (e) Quantification of relative anti-Rh1 band intensities, normalized with the anti-tubulin bands. ANOVA followed by Tukey’s post hoc test was used for statistical analysis. N.s. = not significant. *** $p < 0.0005$. (f) Retinal degeneration as assessed through the percentage of flies retaining intact fluorescent pseudopupils, as assessed with Rh1-GFP. The genotypes are indicated. Male flies were used for the analysis. N numbers for each genotype are as follows: control (*w*^{1118}) = 76, *NorpA*^{EE5}*/Y* = 52, *NorpA*^{EE5}*/Y; Rh1-Gal4/UAS-NECAP*^{WT} = 56, *NorpA*^{EE5}*/Y; Rh1-Gal4/UAS-NECAP*^{G176R} = 31. Log-rank test was used to assess the statistical significance between *NorpA*^{EE5}*/Y* and *NorpA*^{EE5}*/Y; Rh1-Gal4/UAS-NECAP*^{wt}. **** $p < 0.00005$.

bioRxiv preprint doi: https://doi.org/10.64898/2026.06.01.729193; this version posted June 2, 2026. The copyright holder for this preprint (which was not certified by peer review) is the author/funder, who has granted bioRxiv a license to display the preprint in perpetuity. It is made available under a CC-BY 4.0 International license.

Supplemental Figure Legends:

Figure S1: NECAP sequence alignment and the design of the mutant alleles. (a) Sequence alignment of NECAP proteins across species. Human NECAP1, NECAP2, *C. elegans* ncap-1, and the two protein isoforms of *Drosophila* NECAP (products of alternative splicing) are aligned. Key amino acids relevant to this study are shown. The $NECAP^{KO}$ allele used in this study deletes sequences after the 27th amino acid residue in the long protein isoform. The *Drosophila* NECAP protein has high sequence similarity to the human homologs throughout the sequence, including the N-terminal PHear domain and the C-terminal WVQF motif. (b) The design of the $NECAP^{KO}$ allele. (c) PCR validation of the knockout. The regions amplified by PCR are indicated in (b). (d) The design of the $NECAP^{T2A}$ allele. (e) PCR validation of the transposon insertion that disrupts transcripts 3' to the insertion site. The PCR amplified regions are indicated with arrows in (b).

Figure S2: *NECAP* expression assessed by *yw, NECAP*^{T2A}-associated Gal4 driving *UAS-tdTomato* expression. (a) Whole body adult male flies. The fly on the left is a negative control that has an *FM7* chromosome instead of the $NECAP^{T2A}$ allele. (b, b') Adult retina of the genotype *yw, ninaE-GFP, NECAP*^{T2A}*; uas-tdTomato/+* (0 day after eclosion). *ninaE-GFP* expression (green) marks the rhabdomere (apical membrane) of photoreceptor cells. tdTomato fluorescence is detected around those rhabdomeres, where the photoreceptor cell bodies reside. (b') is the tdTomato only channel of the image shown in (b). Scale bar in b represents 10 μm.

bioRxiv preprint doi: https://doi.org/10.64898/2026.06.01.729193; this version posted June 2, 2026. The copyright holder for this preprint (which was not certified by peer review) is the author/funder, who has granted bioRxiv a license to display the preprint in perpetuity. It is made available under a CC-BY 4.0 International license.

Figure S3: Immuno-labeling with Phalloidin shows abnormal retinal organization in *NECAP* mutants exposed to constant light. Confocal microscope images of fixed retina from five-day-old adults reared under constant light. Phalloidin labeling (green) marks rhabdomeres. (a) A control retina (genotype: *yw/w*) shows a regular array of ommatidia, each with seven photoreceptors arranged in a cluster. (b) The *NECAP* homozygous mutant retina (genotype: *yw, NECAP*^{T2A}*/w, NECAP*^{KO}) does not have an organized array of ommatidia. The scale bar in b is 10 μm.
